# Internet use and difficulties in acquiring health resources among older adults with disabilities during the COVID-19 pandemic: a population-based cross-sectional study

**DOI:** 10.1186/s12889-024-17922-2

**Published:** 2024-02-20

**Authors:** Eunjin Yang, Min Jung Kim, Kyung Hee Lee

**Affiliations:** 1 College of Nursing, Gachon University, Incheon, South Korea; 2 Mo-Im Kim Nursing Research Institute, Yonsei University College of Nursing, 50-1 Yonsei-Ro, Seodaemun-Gu, Seoul, 03722 Republic of Korea

**Keywords:** COVID-19, Internet use, Health resources, Older adults, People with disabilities

## Abstract

**Background:**

The various restrictions caused by the COVID-19 pandemic may have worsened the digital divide and health inequality. However, research to ascertain the association between Internet use and difficulties in acquiring health resources among older adults with disabilities is scarce. This study aimed to explore the relationship between Internet use and difficulties in acquiring health resources among older adults with disabilities during the COVID-19 pandemic and explore the associated factors by disability severity.

**Methods:**

Data from the 2020 survey of people with disabilities in South Korea were used. This secondary analysis study included 4,871 older adults aged 55 and above among 7,025 total responders. Complex sample logistic regression analyses were conducted to identify the association between Internet use and difficulties in acquiring health resources during the pandemic.

**Results:**

Only 23.66% of older adults with disabilities used the Internet. Internet non-users were more likely to experience difficulties in obtaining health resources than Internet users. The relationship between Internet non-use and difficulties in acquiring COVID-19-related information (OR 1.57, 95% CI 1.28–1.92) and buying and using personal protective equipment (OR 1.36, 95% CI 1.11–1.65) were statistically significant in the overall sample. Whereas, difficulties with using medical services were not statistically significantly associated with Internet use. Additionally, factors associated with difficulties in acquiring health resources differed by disability severity.

**Conclusions:**

Considering that older adults with disabilities experience triple the burden amid COVID-19 due to old age, disabilities, and the digital divide, policymakers, healthcare professionals, and engineers should aim to narrow the gaps between Internet users and Internet non-users among this population. Narrowing the gaps will make decreasing health gaps and increasing well-being among older adults with disabilities more attainable.

## Background

Along with rapid digital transformation, Internet access has become an important social determinant of health during the COVID-19 pandemic [1]. Social distancing, together with related guidelines, was stipulated by the government as a measure to prevent the spread of the disease [2]. Due to these guidelines, people were more likely to perform various health-related activities as well as social activities, such as Internet banking, shopping, education, work, and leisure activities, through the Internet [3, 4]. This was an effective and efficient way to ensure the continuation of daily life activities even with social distancing. However, the pandemic worsened digital divide-related issues in specific populations [5, 6].

According to the United Nations Secretary-General António Guterres, “the digital divide is now a matter of life and death for people who are unable to access essential health-care information” [7]. The digital divide refers to the gap between those who are connected and those who are not connected to the Internet and related technologies [8]. Older adults with disabilities, who face a two-fold burden due to old age and disabilities, use the internet less compared to people with no disabilities [9]. People with disabilities tend to be less aware of non-face-to-face online services such as subscription, delivery, and other services that became prevalent during the pandemic compared with those with no disabilities [10]. Therefore, older adults with disabilities are the most vulnerable population in terms of experiencing both digital and social exclusion as they are less likely to take advantage of the Internet, although it can augment their quality of life, particularly amid the application of a social-distancing policy [11, 12].

People worldwide experienced tremendous fear due to the non-existence of vaccines and specific treatments in the early phase of the pandemic and thus attempted to seek COVID-19-related health resources [13]. Immediately after the declaration of COVID-19 as a pandemic, health resource-seeking behaviors regarding COVID-19 information peaked worldwide [14]. This information regarded protective behaviors including wearing masks, handwashing, and seeking health knowledge related to vaccines [15]. In this situation, the Korean government provided information on infection rates, hospitalization, vaccination availability, and death through Internet-based platforms [16, 17] and adopted an online pandemic surveillance system [18, 19]. As Internet-based platforms have been widely used to disseminate COVID-19 information and to provide materials related to protective behaviors and remote telehealth services, due to the pandemic, the internet is now considered a more critical and essential tool to acquire health resources than before. Moreover, seeking online COVID-19 information, buying and using personal protective equipment, and using health services are all important health resources and behaviors in this pandemic era.

Evidence has supported an association between Internet use and health outcomes or healthcare use. Internet use was associated with a lower prevalence of chronic conditions and fewer visits to healthcare facilities [20] as well as better physical/cognitive health, well-being, and health behaviors among older adults [21]. People with disabilities who use the Internet were found to perform more physical activities, feel happier, and experience less loneliness than Internet non-users [22]. However, little attention was paid to the effect of Internet use on accessing healthcare resources such as acquiring health information, using personal protective equipment, and healthcare use during COVID-19 among older adults with disabilities. Although older adults with severe impairments are less likely to use ICT such as computer and Internet use compared to those with mild impairments, and the patterns and factors of these gaps could differ by disability severity, researches focused on these issues were scarce. Such guidelines on health and preventive health behaviors are crucial to mitigate the risk of infection among vulnerable populations during the COVID-19 pandemic crisis. To strategize in regard to lessening the health inequality among older adults with disabilities, it is necessary to examine the health impact of Internet use on this specific population in a highly adverse situation such as the COVID-19 pandemic. Therefore, this study aimed to identify the relationship between Internet use and difficulties in the use of health resources among older adults with disabilities during the pandemic.

## Methods

### Data and study participants

This is a population-based cross-sectional study using data from the 2020 11th National Survey on Persons with Disabilities carried out in South Korea. This is an ongoing survey that has been conducted every three years by the Korea Institute for Health and Social Affairs (KIHASA) with funding from the Ministry of Health and Welfare of South Korea. The survey targeted the following 15 types of disabilities defined in the Welfare Act for People with Disabilities: physical disability, disability related to brain lesions, visual impairment, hearing impairment, speech disability, intellectual disability, mental illness, autism spectrum disorder, kidney dysfunction, cardiac dysfunction, respiratory dysfunction, hepatic dysfunction, facial disfigurement, intestinal fistular/urinary fistular, and epilepsy. The goals of the original survey were to estimate the number of people with disabilities, verify the demographic characteristics of people with disabilities nationwide, and evaluate current disability policy.

A two-stage cluster sampling was adopted for this survey. A total of 248 national districts were selected and the survey teams contacted 11,120 eligible participants who were registered as persons with disabilities in the government disability registration system. A total of 7,025 individuals among these 11,120 voluntarily participated in the survey. Regarding the survey procedure, the final survey forms and programs for tablet-assisted personal interviews were determined through a pilot test in September 2019 to assess the questionnaire, participants’ understanding, and response time. The survey was implemented through tablet-assisted personal interviews from October 2020 to February 2021 by trained survey teams. A total of 150 surveyors ranging in age from their 30 to 50 s who had experienced more than 10 instances of participation in face-to-face interviews as surveyors for the national surveys and had survey experience with people with disabilities were selected. In addition, 20 supervisors were selected to monitor and advise the survey process and manage surveyors who were assigned as a team. The survey teams were educated during the course of three days of training, which consisted of two days aimed at understanding the survey and one day focused on learning about the tablet-assisted personal interview program. The trained surveyors visited the homes of participants who volunteered to participate in this survey and conducted tablet-assisted personal interviews. The interview was conducted with self-reports or proxy assessment, and only when the persons with disability could not join the interviews due to reasons such as communication problems, their family proxy responded to the questions. The inclusion criterion for this study was middle-aged and older adults with disabilities, aged 55 and above, considering the premature aging observed in people with long-term disabilities [9]; therefore, of the 7,025 total participants, 4,871 were included in this study. Detailed information regarding the survey process, sampling weight, and survey results were presented in the report on the 2020 National Survey on Persons with Disabilities [23].

### Variables

#### Dependent variables

Difficulties in acquiring health resources during the COVID-19 pandemic were investigated using the following three questions: “Did you face any difficulty acquiring information regarding COVID-19 prevention guidelines and the prevalence of the infection?” “Did you face any difficulty buying and using personal protective equipment (e.g., face masks, sanitizers)?” and “During the COVID-19 pandemic, to what extent did you experience difficulty using medical services (e.g., hospitals, pharmacies) compared with before the pandemic?” The possible answers were categorized as “no difficulties,” “moderate difficulties,” and “severe difficulties.” For the statistical analyses, the responses reflecting moderate and severe difficulties were combined into one category indicating “having difficulties” to make the variable a binary.

#### Independent variables

The independent variable was whether the participants used the Internet, and it was assessed using a single question: “Do you use the Internet?” The response was either “yes” or “no.” The participants who answered “yes” were considered part of the Internet users group, while those who answered “no” were categorized into the Internet non-users group.

#### Covariates

Demographic characteristics such as types of disabilities, level of disability (mild, severe), age, gender, marital status, self-reported economic status (lower class, middle class, upper class, for which respondents self-reported whether they belonged to a certain group regarding their economic status), education (none, high school or below, college or above), living arrangements (living alone, living with others), living area (urban, rural), and chronic disease (yes, no) were considered as covariates. These factors were revealed as factors related to Internet use in a previous study [24].

### Statistical analysis

The 2020 National Survey on Persons with Disabilities used a complex sample design method; therefore, we used the SPSS complex sample data analysis procedure as it was the appropriate method for data with weights. Post-stratification adjustments were made to correct for sample bias and to represent the characteristics of people with disabilities in South Korea. This sample weight was provided by the KIHASA [23]. Inverse probability weighting adjustment and raking ratio estimation were conducted based on the information on disability types and severity in each national district. More detailed information on the sample weight calculation can be found in the report on the 2020 National Survey on Persons with Disabilities [23]. Descriptive statistics were calculated to explore the participants’ characteristics. Unweighted numbers of observations and weighted percentages for categorical variables were presented through complex sample frequency analysis. Weighted means and standard errors for continuous variables were presented through complex samples descriptive analysis considering the sample weights. Complex sample multivariate logistic regression analyses were performed to examine the association between Internet use and difficulties in acquiring health resources during the COVID-19 pandemic. The findings are presented as odds ratios (ORs) with 95% confidence intervals (95% CI). Pseudo R^2^ was reported for each model. All analyses were conducted with the SPSS Statistics for Windows (version 26.0; IBM Corp).

### Ethical considerations

Data were publicly available, and the researchers followed regulations and guidelines when using the data. This study was exempted from ethical approval by the Institutional Review Board of the researchers’ affiliated institution (No.4-2022-0635).

## Results

### Participants’ demographics

Descriptive statistics for the participants’ demographics are provided in Table 1. Approximately half of the participants (51.1%) were people with a physical disability. Among the participants, 28.7% had severe disabilities and 71.3% had mild disabilities. Different demographic characteristics were observed between the two groups by disability severity except for living area. The mean age of persons with mild disabilities (71.79 ± 0.23) was higher than that of persons with severe disabilities (69.11 ± 0.24). The proportion of male was higher in severe disability groups compare to mild disability groups (*P* =.001) and people who married was higher in mild disability groups compared to severe disability groups (*P* =.025). The people in lower economic class was higher in severe groups (*P* <.001) and participants with no education were higher in mild groups (*P* =.031). The rate of participants living alone was higher in mild groups (*P* =.015); whereas, the rate of participants with chronic disease was higher in severe groups (*P* <.001).

**Table 1:** Demographic characteristics of the participants (*N* = 4871)

Variables	Total (*N* = 4871)		Severe disability (*n* = 2141)		Mild disability (*n* = 2730)		*p* value
**Types of disabilities, n (%)** ^a^							
Physical disability	1,419	(51.10)	468	(31.89)	951	(58.81)	< 0.001
Brain lesion	640	(9.62)	339	(19.33)	301	(5.72)	
Visual impairment	600	(9.83)	202	(6.51)	398	(11.17)	
Hearing impairment	893	(19.0)	278	(13.94)	620	(21.03)	
Mental disability	246	(3.84)	246	(13.42)	0	(0)	
Internal disability	881	(5.64)	495	(12.73)	386	(2.79)	
Speech disability	145	(0.85)	92	(1.89)	53	(0.43)	
Facial disfigurement	47	(0.11)	26	(0.27)	21	(0.04)	
**Age (years), mean (SE)**	71.02	(0.18)	69.11	(0.24)	71.79	(0.23)	< 0.001
**Gender, n (%)**							
Male	2,763	(54.01)	1259	(57.84)	1504	(52.46)	0.001
Female	2,108	(45.99)	882	(42.16)	1226	(47.54)	
**Marital status, n (%)** ^a^							
Married	2,783	(56.29)	1179	(53.61)	1604	(57.36)	0.025
Others^b^	2,088	(43.71)	962	(46.39)	1126	(42.64)	
**Self-reported economic status, n (%)** ^a^							
Lower class	3,445	(70.87)	1578	(75.17)	1867	(69.15)	< 0.001
Middle class	1,378	(27.92)	547	(24.04)	831	(29.48)	
Upper class	48	(1.20)	16	(0.80)	32	(1.37)	
**Education, n (%)** ^a^							
None	476	(11.57)	199	(10.27)	277	(12.09)	0.031
High school or below	3,936	(80.10)	1730	(79.94)	2206	(80.17)	
College or above	459	(8.33)	212	(9.80)	247	(7.75)	
**Living arrangements, n (%)** ^a^							
Living alone	1,450	(31.49)	611	(28.79)	839	(32.58)	0.015
Living with others	3,421	(68.51)	1530	(71.21)	1891	(67.42)	
**Living area, n (%)** ^a^							
Urban	2,252	(39.92)	953	(38.08)	1299	(40.65)	0.109
Rural	2,619	(60.08)	1188	(61.92)	1431	(59.35)	
**Chronic disease, n (%)** ^a^							
Yes	4,044	(80.73)	1842	(84.42)	2202	(79.25)	< 0.001
No	827	(19.27)	299	(15.58)	528	(20.75)	
**Internet use, n (%)** ^a^							
Yes	1,195	(23.66)	477	(21.56)	718	(24.50)	0.04
No	3,676	(76.34)	1664	(78.44)	2012	(75.50)	
**Reasons for no internet use, n (%)** ^a^	**Total** **(n = 3676)**		**Severe** **disability** **(n = 1664)**		**Mild ** **disability** **(n = 2012)**		
Economic burden or not having devices	238	(7.01)	96	(6.32)	142	(7.29)	0.52
No need	2,171	(58.25)	1015	(59.72)	1156	(57.64)	
Complex to use or hard to learn to use	852	(23.08)	377	(22.85)	475	(23.18)	
Lack of awareness of how the Internet can be used	330	(9.87)	137	(9.03)	193	(10.22)	
Other	85	(1.79)	39	(2.08)	46	(1.67)	

^a^ Numbers are unweighted and percentages are weighted

^b^ Others: widowed, divorced, separated, single

Regarding Internet use, only 23.66% of overall participants were Internet users and the rate of Internet use was higher in mild groups (24.50% in mild disability vs. 21.56% in severe disability, *P* =.04). Most Internet non-users (58.25%) responded that Internet use was not required. Complexity and difficulty in learning to use the Internet as well as a lack of awareness of how the Internet can be used were the second and third most-common responses, respectively, regarding the reasons for not using the Internet in both groups.

### Participants’ characteristics according to the difficulties in acquiring health resources

The characteristics of the participants, according to their responses in each domain concerning difficulties in acquiring health resources, are presented in Table 2. In terms of difficulties in acquiring COVID-19 information, significant statistical differences were observed in age (*P* =.004), gender (*P* =.009), marital status (*P* <.001), self-reported economic status (*P* <.001), educational level (*P* <.001), living arrangement (*P* <.001), living area (*P* <.001), and internet use (*P* <.001), with the exception of the types of disabilities and chronic disease.

**Table 2:** Characteristics of the participants according to the responses to difficulties in acquiring health resources (*N* = 4871)

Variables	Difficulties in ^a^								
	Acquiring COVID-19 information			Buying and using personal protective equipment			Using medical services		
	Yes (n = 2140)	No (n = 2730)	*P* Value	Yes (n = 2172)	No (n = 2699)	*P* Value	Yes (n = 2563)	No (n = 2308)	*P* Value
**Types of disabilities, n (%)** ^a^			0.053			0.167			< 0.001
Physical disability	634(51.76)	785(50.52)		655(51.77)	764(50.50)		723(49.33)	696(53.13)	
Brain lesion	292(10.04)	348(9.26)		300(10.16)	340(9.14)		401(11.64)	239(7.30)	
Visual impairment	275(9.63)	325(10.01)		284(9.95)	316(9.73)		314(9.69)	286(9.99)	
Hearing impairment	382(18.40)	511(19.52)		385(18.26)	508(19.66)		449(18.68)	444(19.37)	
Mental disability	131(4.48)	115(3.30)		124(4.09)	122(3.63)		141(4.19)	105(3.45)	
Internal disability	340(4.82)	541(6.36)		338(4.89)	543(6.31)		422(5.33)	459(6.00)	
Speech disability	65(0.77)	80(0.92)		66(0.82)	79(0.88)		90(1.05)	55(0.62)	
Facial disfigurement	21(0.11)	26(0.11)		20(0.08)	27(0.14)		23(0.10)	24(0.12)	
**Age (years), mean (SE)**	71.57(0.27)	70.55(0.23)	0.004	71.33(0.26)	70.75(0.24)	0.101	72.05(0.25)	69.84(0.24)	< 0.001
**Gender, n (%)**			0.009			0.527			< 0.001
Male	1164(51.50)	1599(56.16)		1813(56.94)	2386(58.51)		1384(50.56)	1379(58.95)	
Female	976(48.50)	1132(43.84)		1269(43.06)	1557(41.49)		1179(49.44)	929(42.05)	
**Marital status, n (%)** ^a^			< 0.001			0.016			< 0.001
Married	1127(52.00)	1656(60.00)		1447(48.36)	1929(50.95)		1394(52.23)	1389(60.94)	
Others^b^	1013(48.00)	1075(40.00)		1635(51.64)	2014(49.05)		1169(47.77)	919(39.06)	
**Self-reported economic status, n (%)** ^a^			< 0.001			< 0.001			< 0.001
Lower class	1667(77.57)	1778(65.09)		2311(75.20)	2533(64.47)		1925(75.18)	1520(65.93)	
Middle class	454(21.20)	924(33.76)		744(23.78)	1370(34.49)		614(23.61)	764(32.87)	
Upper class	19(1.30)	29(1.15)		27(1.01)	40(1.03)		24(1.21)	24(1.20)	
**Education, n (%)** ^a^			< 0.001			< 0.001			< 0.001
None	254(14.20)	222(9.29)		283(10.69)	286(7.93)		290(13.79)	186(9.01)	
High school or below	1735(79.70)	2201(80.45)		2441(78.14)	2982(75.26)		2067(79.50)	1869(80.79)	
College or above	151(6.10)	308(10.26)		358(11.17)	675(16.81)		206(6.71)	253(10.20)	
**Living arrangements, n (%)** ^a^			< 0.001			0.007			0.001
Living alone	728(35.45)	722(28.08)		849(29.03)	960(25.63)		808(34.01)	642(28.61)	
Living with others	1412(64.55)	2009(71.92)		2233(71.97)	2983(74.37)		1755(65.99)	1666(71.39)	
**Living area, n (%)** ^a^			< 0.001			< 0.001			< 0.001
Urban	893(35.97)	1359(43.32)		1290(35.39)	1976(43.81)		1077(36.73)	1175(43.57)	
Rural	1247(64.03)	1372(56.68)		1792(64.61)	1967(56.19)		1486(63.27)	1133(56.43)	
**Chronic disease, n (%)** ^a^			0.063			0.168			0.138
Yes	1758(79.28)	2286(82.98)		2247(70.00)	2884(71.17)		2155(81.73)	1889(79.58)	
No	382(20.72)	445(18.02)		835(30.00)	1059(28.83)		408(18.27)	419(20.42)	
**Internet use, n (%)** ^a^			< 0.001			< 0.001			< 0.001
Yes	381(17.87)	814(28.65)		971(31.49)	1617(40.76)		512(19.06)	683(28.93)	
No	1759(82.13)	1917(71.35)		2111(68.51)	2326(59.24)		2051(80.94)	1625(71.07)	

^a^ Numbers are unweighted and percentages are weighted

^b^ Others: widowed, divorced, separated, single

Characteristics including marital status (*P* =.016), self-reported economic status (*P* <.001), educational level (*P* <.001), living arrangements (*P* =.007), living area (*P* <.001), and internet use (*P* <.001) were statistically different between the two groups, one with and the other without difficulties in buying using protective personal equipment.

Regarding the difficulties in using medical services, all characteristics, such as types of disabilities, age (*P* <.001), gender (*P* <.001), marital status (*P* <.001), self-reported economic status (*P* <.001), educational level (*P* <.001), living arrangement (*P* =.001), living area (*P* <.001), and internet use (*P* <.001), were statistically different between two groups, except for chronic disease.

### Multivariate logistic regression model of difficulties in acquiring health resources

Table 3 shows the logistic regression results. Odds ratios (OR) were calculated for all independent variables and acquiring health resources. The difficulties in acquiring COVID-19 information were higher among Internet non-users (OR = 1.57, 95% CI 1.28–1.92), people with severe disabilities (OR = 1.19, 95% CI 1.00–1.38), people with no education (OR = 1.52, 95% CI 1.06–2.19), and living in rural areas (OR = 1.31, 95% CI 1.13–1.52). The odds of facing difficulties in buying and using personal protective equipment were also higher in Internet non-users (OR = 1.36, 95% CI 1.11–1.65), people with severe disabilities (OR = 1.24, 95% CI 1.07–1.43), and those living in rural areas (OR = 1.32, 95% CI 1.14–1.53), whereas education showed no association. Person with severe disability (OR = 1.27, 95% CI 1.09–1.47), older age (OR = 1.02, 95% CI 1.01–1.03), and living in a rural area (OR = 1.33, 95% CI 1.15–1.54) were associated with greater difficulties in using medical services, whereas Internet use was not associated with using medical services.

**Table 3:** Logistic regression model of perceived difficulties in acquiring health resources (*N* = 4871)

Variables	Difficulties in ^a^								
	Acquiring COVID-19 information			Buying and using personal protective equipment			Using medical services		
	Model 1: crude		Model 2: logistic	Model 1: crude		Model 2: logistic	Model 1: crude		Model 2: logistic
	**OR** ^d^ ** (95% CI)**		**OR** ^d^ ** (95% CI)**	**OR**^d^ ** (95% CI)**		**OR (95% CI)**	**OR**^d^ **(95% CI)**		**OR (95% CI)**
**Internet use**									
No	1.85(1.55–2.20)***		1.57 (1.28–1.92)***	1.51(1.27–1.79)***		1.36 (1.11–1.65)**	1.73(1.46–2.05)***		1.20 (0.99–1.46)
Yes (Reference)	1		1	1		1			1
**Types of disabilities**									
Physical disability			0.90 (0.38–2.15)			1.98 (0.88–4.50)			1.03 (0.43–2.49)
Brain lesion			0.89 (0.37–2.14)			1.97 (0.86–4.49)			1.65 (0.68–4.02)
Visual impairment			0.81 (0.34–1.96)			1.93 (0.84–4.42)			1.03 (0.42–2.53)
Hearing impairment			0.74 (0.31–1.76)			1.66 (0.73–3.77)			0.89 (0.37–2.17)
Mental disability			0.89(0.36–2.21)			1.62(0.68–3.83)			1.09(0.43–2.76)
Internal disability			0.71(0.30–1.70)			1.50(0.66–3.41)			1.02(0.42–2.47)
Speech disability			0.70(0.27–1.80)			1.67(0.67–4.13)			1.82(0.69–4.80)
Facial disfigurement (Reference)			1			1			1
**Level of disability**									
Severe			1.19 (1.00–1.38)*			1.24 (1.07–1.43)**			1.27 (1.09–1.47)**
Mild (Reference)			1			1			1
**Age**			1.00 (1.00–1.01)			1.00 (0.99–1.01)			1.02 (1.01–1.03)***
**Gender**									
Female			1.00 (0.86–1.18)			0.93 (0.79–1.09)			1.13 (0.96–1.32)
Male (Reference)			1			1			1
**Living arrangements**									
Living alone			1.13 (0.91–1.41)			1.09 (0.88–1.35)			0.94 (0.76–1.17)
Living with others (Reference)			1			1			1
**Marital status**									
Married			0.96 (0.77–1.19)			1.01 (0.82–1.25)			0.84 (0.68–1.03)
Other (Reference)			1			1			1
**Education**									
None			1.52 (1.06–2.19)*			1.40 (0.98–2.01)			1.28 (0.90–1.84)
High school or below			1.24 (0.94–1.64)			1.22 (0.92–1.60)			1.17(0.90–1.52)
College or above (Reference)			1			1			1
**Self-reported economic status**									
Lower class			0.93 (0.44–1.94)			1.02 (0.49–2.13)			1.06 (0.49–2.29)
Middle class			0.54 (0.26–1.14)			0.66 (0.32–1.38)			0.72 (0.33–1.56)
Upper class (Reference)			1			1			1
**Chronic disease**									
Yes			0.74 (0.64–0.90)**			0.82 (0.68–1.00)*			0.95 (0.79–1.15)
No (Reference)			1			1			1
**Living area**									
Rural			1.31 (1.13–1.51)***			1.32 (1.14–1.53)***			1.33 (1.15–1.54)***
Urban (Reference)			1			1			1
**Cox and Snell R** ^**2**^	0.046			0.029			0.045		
**Nagelkerke R** ^**2**^	0.061			0.038			0.060		

^a^ There were 2140 (unweighted number) observations for difficulties in acquiring COVID-19 information, 2172 (unweighted number) for difficulties in buying and using personal protective equipment, and 2563 (unweighted number) for difficulties in using medical services

^b^ B: coefficient

^c^ SE: Standard error

^d^ OR, Odds ratio derived from multivariate logistic regression

^*^ Significant at the *P* <.05 level

^**^ Significant at the *P* <.01 level

^***^ Significant at the *P* <.001 level

### Factors related to difficulties in acquiring health resources by disability severity

The factors associated with difficulties in acquiring health resources amid the COVID-19 pandemic differed by disability severity and are presented in Table 4. Internet use and living areas were associated with difficulties acquiring COVID-19 information in both severe and mild disability groups. Among the demographic characteristics, persons with chronic disease were less likely to have difficulties only in mild disability groups (OR = 0.69, 95% CI 0.54–0.87).

**Table 4:** Logistic regression model of perceived difficulties in acquiring health resources by disability severity (*N* = 4871)

Variables	Difficulties in					
	Acquiring COVID-19 information		Buying and using personal protective equipment		Using medical services	
	Severe disability	Mild disability	Severe disability	Mild disability	Severe disability	Mild disability
	OR^a^ (95% CI)	OR (95% CI)	OR (95% CI)	OR (95% CI)	OR (95% CI)	OR (95% CI)
**Internet use**						
No	1.79(1.36–2.36)***	1.48(1.14–1.93)**	1.37(1.05–1.80)**	1.35(1.05–1.75)**	1.31(1.00–1.71)	1.16(0.90–1.49)
Yes (Reference)	1	1	1	1	1	1
**Types of disabilities**						
Physical disability	1.02(0.31–3.39)	0.66(0.28–1.55)	3.25(1.08–9.79)**	0.78(0.34–1.79)	0.99(0.32–3.12)	1.33(0.55–3.24)
Brain lesion	0.98(0.29–3.27)	0.68(0.28–1.65)	2.90(0.96–8.78)	0.85(0.36–2.00)	1.34(0.42–4.25)	2.55(1.02–6.38)*
Visual impairment	1.23(0.36–4.21)	0.56(0.23–1.33)	3.43(1.11–10.60)**	0.75(0.32–1.73)	1.11(0.34–3.58)	1.31(0.53–3.24)
Hearing impairment	0.96(0.29–3.22)	0.52(0.22–1.24)	2.81(0.92–8.55)	0.65(0.28–1.49)	0.90(0.28–2.87)	1.13(0.46–2.77)
Mental disability	0.99(0.29–3.39)	–	2.64(0.85–8.18)	–	0.96(0.30–3.10)	–
Internal disability	0.80(0.24–2.66)	0.48(0.15–1.49)	2.33(0.77–7.05)	0.61(0.26–1.42)	0.94(0.30–2.96)	1.34(0.54–3.31)
Speech disability	0.80(0.23–2.87)	1.00(0.77–1.45)	2.88(0.88–9.42)	0.56(0.19–1.67)	1.43(0.42–4.89)	3.07(0.97–9.72)
Facial disfigurement (Reference)	1	1	1	1	1	1
**Age**	1.00(0.99–1.02)	1.01(0.99–1.02)	1.01(0.99–1.02)	1.00(0.99–1.01)	1.01(1.00–1.02)	1.03(1.01–1.04)^***^
**Gender**						
Female	0.87(0.70–1.08)	1.07(0.86–1.31)	0.87(0.70–1.07)	0.95(0.77–1.17)	1.20(0.97–1.49)	1.09(0.89–1.34)
Male (Reference)	1	1	1	1	1	1
**Living arrangements**						
Living alone	0.86(0.65–1.15)	1.29(0.96–1.75)	0.85(0.64–1.13)	1.24(0.92–1.66)	0.92(0.69–1.23)	0.92(0.69–1.24)
Living with others (Reference)	1	1	1	1	1	1
**Marital status**						
Married	0.78(0.59–1.02)	1.07(0.79–1.44)	0.85(0.65–1.11)	1.11(0.82–1.49)	0.95(0.73–1.25)	0.79(0.59–1.05)
Other (Reference)	1	1	1	1	1	1
**Education**						
None	1.52(0.93–2.47)	1.54(0.94–2.50)	1.72(1.06–2.79)**	1.30(0.80–2.10)	0.94(0.58–1.53)	1.45(0.90–2.33)
High school or below	1.28(0.89–1.83)	1.25(0.85–1.84)	1.34(0.94–1.91)	1.18(0.81–1.72)	1.00(0.71–1.43)	1.26(0.88–1.81)
College or above (Reference)	1	1	1	1	1	1
**Self-reported economic status**						
Lower class	0.93(0.27–3.16)	0.94(0.39–2.23)	0.63(0.20–2.03)	1.15(0.48–2.77)	1.54(0.50–4.76)	0.98(0.39–2.50)
Middle class	0.58(0.17–2.00)	0.54(0.22–1.29)	0.47(0.15–1.51)	0.71(0.30–1.73)	1.03(0.33–3.21)	0.68(0.26–1.73)
Upper class (Reference)	1	1	1	1	1	1
**Chronic disease**						
Yes	0.93(0.70–1.24)	0.69(0.54–0.87)**	0.95(0.71–1.26)	0.79(0.62–1.00)	0.87(0.66–1.17)	0.97(0.76–1.22)
No (Reference)	1	1	1	1	1	1
**Living area**						
Rural	1.25(1.03–1.53)*	1.32(1.09–1.60)**	1.25(1.03–1.53)**	1.34(1.11–1.62)**	1.21(0.99–1.47)	1.39(1.15–1.67)***
Urban (Reference)	1	1	1	1	1	1
**Cox and Snell R** ^**2**^	0.041	0.049	0.025	0.031	0.023	0.051
**Nagelkerke R** ^**2**^	0.055	0.065	0.033	0.041	0.031	0.069

^a^ OR, Odds ratio derived from multivariate logistic regression

^*^ Significant at the *P* <.05 level

^**^ Significant at the *P* <.01 level

^***^ Significant at the *P* <.001 level

Regarding difficulties in buying and using personal protective equipment, higher odds ratios were observed in non-Internet users and persons living in rural areas in both groups. However, among various types of disabilities, the people with physical disability (OR = 3.25, 95% CI 1.08–9.79) and persons with visual impairment (OR = 3.43, 95% CI 1.11–10.60) had statistically higher odds ratios in the severe groups. Additionally, participants with no education were statistically significant only in severe groups (OR = 1.72, 95% CI 1.06–2.79).

Participants living in rural areas were also more likely to have difficulties in using medical services. However, this association was statistically significant only in mild groups (OR = 1.39, 95% CI 1.15–1.67). Age (OR = 1.03, 95% CI 1.01–1.04) and types of disabilities also were significant only in mild groups. Among the type of disabilities, brain lesions ware statistically significant (OR = 2.55, 95% CI 1.02–6.38).

## Discussion

This study examined the association between Internet use and perceived difficulties in acquiring health resources such as COVID-19 information, personal protective equipment, and medical services use among older adults with disabilities during the COVID-19 pandemic. The study focused on Internet use and the COVID-19 pandemic era because the latter may have worsened the digital divide between Internet non-users and users specifically pertaining to public health. After controlling for the covariates in the logistic regression model, Internet use was associated with difficulties in acquiring COVID-19 information and using personal protective equipment, while no association was observed for medical services use in overall participants.

Internet use was found to be the most powerful factor associated with difficulties in acquiring COVID-19 information. Moreover, a previous study reported that access to the Internet was an important factor in determining accessibility to telehealth services during the COVID-19 pandemic among older adults [25]. Our results and the collective results of previous studies indicate that Internet non-users among older adults with disabilities may have been excluded from access to necessary health information during the COVID-19 pandemic. This exclusion can significantly impact their overall health status because having adequate access to health information and healthcare is critical for those living with disability. Although the COVID-19 pandemic is not as dire as it once was, the Internet remains a major health information source, and COVID-19 information is still an essential health resource. Digital transformation during the pandemic has already changed services in care sectors in terms of the use of several technologies, such as artificial intelligence, the Internet of things, and robotics. These changes are expected to continue in the present as well as in the post-COVID-19 era. Therefore, efforts to narrow the digital divide in terms of issues including Internet use, skills, and tangible outcomes that are closely related to health concerns are needed to ensure that older adults with disability reap health benefits from the Internet and new technology. The Korean government adopted a communication rate reduction and exemption system, along with the distribution of devices, for vulnerable classes as these efforts. The government expects these measures to increase Internet accessibility [26]. However, over one-third of vulnerable persons are not supported by the system because they are unaware of this provision [27]. Therefore, promoting it through active campaigns or adopting an automated supporting system is warranted after establishing the policy to support vulnerable classes.

The results of this study indicate that Internet non-users among older adults with disabilities had difficulty buying and using personal protective equipment during the pandemic. South Korea as well as the entire world faced public mask shortages in the early stage of the pandemic. During that time, the South Korean government increased mask production via many approaches [28] and shared real-time data on mask supplies at pharmacies [29]. Therefore, people sought information on where they could obtain face masks via the Internet and then visited the pharmacies to buy masks. Governments as well as private sectors attempted to provide people easy access to personal protective equipment via Internet-based platforms [30]. Sharing real-time information regarding face mask supplies was convenient and effective; however, our study results indicate that Internet non-users among older adults with disabilities could have been excluded from these services and had difficulty buying these items. During the pandemic, people with disabilities had higher odds of death involving COVID-19 compared with the general population, and these trends remained unchanged during the first, second, and third [31]. In addition to individuals’ poor baseline health conditions, these outcomes can be partially explained by the low rates of Internet use among older adults with disabilities, and Internet non-users’ difficulty adhering to protective behaviors such as buying and using personal protective equipment. Previous studies have suggested that obtaining Internet health information influenced patients’ compliance with experts’ recommendations [32], and health information-seeking behaviors were associated with COVID-19 preventive behaviors [33]. Although our study only investigated Internet use rather than health information-seeking behaviors, considering that the Internet-based health information platforms, including social media, are currently among the most frequently used health information sources, Internet non-users among older adults with disabilities may find it difficult to comply to COVID-19 protocols.

The association between Internet use and the utilization of medical services was not statistically significant among overall participants. Sub-group analyses also revealed no significant differences in both groups. This might be because most appointments for medical services are still made through telephone consulting services, except in a few tertiary hospitals that actively use online appointment services. Therefore, in South Korea, those who need medical services can easily avail themselves of these services without relying on online resources.

Regarding the reason for not using the Internet, 23.08% of Internet non-users answered that it was difficult to learn how to use the Internet, and 7.0% said that it was an economic burden in this study results. A lack of knowledge on how the Internet can be used (9.87%) was another reason. Previous studies have reported that a lack of proficiency in using the Internet as well as its affordability were the main barriers to sustained Internet use among low-income older adults [34]. Therefore, in addition to the Korean governments’ communication rate reduction and exemption systems for vulnerable population, a well-designed government-led digital literacy support program targeting older adults with disabilities is required to lessen the health impact of the digital divide and to better prepare this target population for future pandemics [35].

Among the covariates, this study found that older adults with severe disabilities were more likely to experience difficulties in acquiring COVID-19 information, obtaining and using personal protective equipment, and using medical services compared with those with mild disabilities in overall participants. As those with severe impairments use the Internet at a diminished rate, and disability severity was negatively associated with acquiring health resources, sub-group analyses were conducted to verify the factors of these difficulties by disability severity. As a result, those with chronic disease were less likely to have difficulties in acquiring COVID-19 information in mild groups. This might be because those with a mild disability, who are more active in social activities than those with severe disabilities, could easily get health information. Specifically, health concerns regarding chronic diseases among those with chronic diseases may have led them to actively seek health information both before and after the pandemic [36].

Regarding the types of disabilities, brain lesions were associated with difficulties in the use of medical services in mild groups, and physical disability and visual impairment increased the likelihood of experiencing difficulties in using personal protective equipment in the severe groups. Brain lesions in particular presented the highest odds ratio among all variables regarding difficulty using medical services in mild disability groups. Therefore, healthcare providers should consider disability types and severity to avoid exclusion from critical medical services during a pandemic regardless of Internet use. These strategies may include establishing databases and systems to evaluate the appropriateness of delivering services to responding to the COVID-19 situation. Furthermore, delivery of COVID-19 health information through appropriate materials and methods considering the individuals’ preferences and acceptance as well as support for personal protective equipment are needed.

Living areas were also associated with difficulties in acquiring health resources across all domain. During the pandemic, rural regions were highly susceptible to COVID-19, which may have been due to their tendency to have a larger older population and a lack of services and labor [37]. Several studies have elucidated the differences the COVID-19 cases, mortality, and preventive behaviors between urban and rural [38, 39], and our study results add evidence regarding the gap in acquiring health resources in addition to known gaps between urban and rural areas.

### Strengths and limitations

This study used national data representing the characteristics of older people with disabilities in South Korea. Although there were many barriers to conducting surveys during the pandemic, this was managed by a large-scale survey company and the KIHASA, a government-supported research institute for health and welfare policy in South Korea. Therefore, the survey results were reliable. However, this study has limitations. First, several variables were assessed through self-reported questionnaires using single-item measures rather than validated tools. For example, economic status was measured as an assessment of the participants’ perceptions of whether they belong to specific economic classes rather than using objective measurement. Internet usage as an independent variable was also assessed using simple questions with binary answers. Second, the overall participation rate of this survey was only about 63.2%, because of the pandemic, which is less than previous survey’s participation rate (81.9% in 2017). Low participation rate could lead to concern about the representativeness of the disability sample. Third, the interview was conducted with both self-reports and proxy interviews. Approximately 4.5% of the participants answered by proxy, due to their inability to communicate because of their disability. Proxy responses could get results out of accordance with thoughts of the disabled participants. Fourth, the survey included Internet users and non-users; therefore, the exact roles of the Internet in decreasing participants’ difficulties in relation to healthcare could not be explained with precision, and we were not able to ascertain a causal relationship. Future longitudinal studies including the purpose of Internet use such as seeking health information, using health care applications, and applying acquired health information to manage health, will be better able to explain this relationship.

## Conclusions

This study shows that Internet non-users among older adults with disabilities were more likely to have difficulty in obtaining COVID-19 information and using personal protective equipment. Older adults with disabilities face the threefold burden of old age, disabilities, and the digital divide amid the COVID-19 pandemic. Considering the vulnerabilities among this population, policymakers, healthcare professionals, and engineers should focus on narrowing the gaps between Internet users and non-users among older adults with disabilities. This will better ensure improvement of the health and well-being of older adults with disabilities.

## Data Availability

The data can be made available on request to corresponding author.

## Abbreviations

KIHASA: Korea Institute for Health and Social Affairs
OR: Odds ratios
